# Para-perirenal fat thickness is associated with reduced glomerular filtration rate regardless of other obesity-related indicators in patients with type 2 diabetes mellitus

**DOI:** 10.1371/journal.pone.0293464

**Published:** 2023-10-26

**Authors:** Sunan Xu, Junqing Ma, Yongze Zheng, Ruichen Ren, Wenting Li, Wei Zhao, Yu Ma, Tao Zhou, Yang Zhang

**Affiliations:** 1 Department of Radiology, Qilu Hospital of Shandong University, Jinan, China; 2 Department of Radiology, Shandong Rongjun General Hospital, Jinan, China; 3 Department of Radiology, Tai’an First People’s Hospital, Tai’an, Shandong, China; The University of the West Indies, JAMAICA

## Abstract

**Purpose:**

To investigate the relationship between estimated glomerular filtration rate (eGFR) and para-perirenal fat thickness in comparison with other indices of adiposity in type 2 diabetes mellitus (T2DM).

**Methods:**

This single-center, retrospective and cross-sectional study evaluated 337 patients with T2DM. The obesity-related indicators including height, weight, body surface area (BSA), body mass index (BMI), waist circumference (WC), waist-to-hip ratio (WHR), para-perirenal fat thickness (PRFT), total abdominal fat (TAF), subcutaneous adipose tissue (SAT), visceral adipose tissue (VAT). eGFR was calculated by CKD-EPI equation. The correlation between eGFR and obesity-related indicators was performed by pearson or spearman correlation analysis and multivariate linear regression.

**Results:**

337 subjects (mean age, 60.2 ± 11.6 years; 195 males, 57.9%) were evaluated. eGFR was negatively correlated with height, weight, BMI, PRFT, TAF, SAT, and VAT, among which the correlation between eGFR and PRFT was the strongest (r = -0.294, p< 0.001). eGFR remained the strongest correlation with PRFT in the subgroup separated by sex (r = -0.319 in the male subgroup, and -0.432 in the female subgroup, respectively, p < 0.001). Age and PRFT were the independent predictive factors for eGFR. PRFT was the best predictor of chronic kidney disease (CKD) in T2DM (AUC = 0.686, p = 0.001, 95% CI: 0.582–0.791). CKD in T2DM can be predicted well by linking age with PRFT (AUC = 0.708, p<0.001, 95% CI = 0.605–0.812).

**Conclusions:**

PRFT is more closely related to glomerular filtration rate than other obesity-related indicators in T2DM. The model combining age with PRFT could predict CKD in T2DM well.

## Introduction

Diabetes mellitus is a metabolic disease characterized by chronic elevation of blood glucose levels caused by inadequate or defective insulin secretion. Chronic kidney disease (CKD) is an important complication of diabetes mellitus [1]. The high morbidity and mortality of diabetic nephropathy (DN) cause a serious economic burden [2, 3]. Obesity is one of the most important risk factors for type 2 diabetes mellitus (T2DM) [4]. Recent epidemiological studies have shown that obesity is an independent risk factor for CKD. The potential mechanisms include diabetes, hypertension, insulin resistance and direct cellular effects such as inflammatory, lipotoxic and hemodynamic factors [5–8]. Previous studies have addressed the relationship between the traditional indicators of obesity and chronic kidney disease, such as body mass index (BMI), waist circumference (WC), and waist-to-hip ratio (WHR) [9, 10]. However, it is difficult for these traditional factors to distinguish adiposity from other tissue components and to clarify different fat distributions. Moreover, they are susceptible to age, race, and physical activity level [11, 12]. Many new methods have emerged in clinical practice and scientific research to assess adipose tissue, especially with the development of medical imaging techniques such as ultrasound (US), computed tomography (CT), magnetic resonance imaging (MRI), and dual-energy x-ray absorptiometry (DXA) [13–16].

Perirenal fat is a special type of visceral fat located in the retroperitoneal space. It surrounds the kidney, separated from the renal parenchyma and pararenal fat by the fibrous capsule and renal fascia. It has intact blood supply, innervation, and lymphatic drainage [17]. In addition to providing mechanical support to the kidney, perirenal fat is an important endocrine organ that produces and secretes cytokines or adipokines [17, 18]. The specific anatomical connection of perirenal fat with the kidney and its endocrine function make it possible to participate in the development of chronic kidney disease [14, 19]. The study by Geraci G et.al showed that PUFT (perirenal fat thickness measured by ultrasound) can predict chronic kidney disease more accurately in hypertensive patients [14]. The studies on patients with T2DM revealed a negative independent correlation between PRFT and eGFR [19, 20]. No study is available to comprehensively assess estimated glomerular filtration rate (eGFR) and para-perirenal fat thickness in comparison with other indices of adiposity.

Our study aimed to investigate the relationship between eGFR and PRFT in patients with T2DM, in comparison with traditional anthropometric indices of adiposity and abdominal fat content measured by CT, which has not been demonstrated local mechanical or paracrine action on the kidney yet.

## Materials and methods

### Subjects

**Fig 1:**
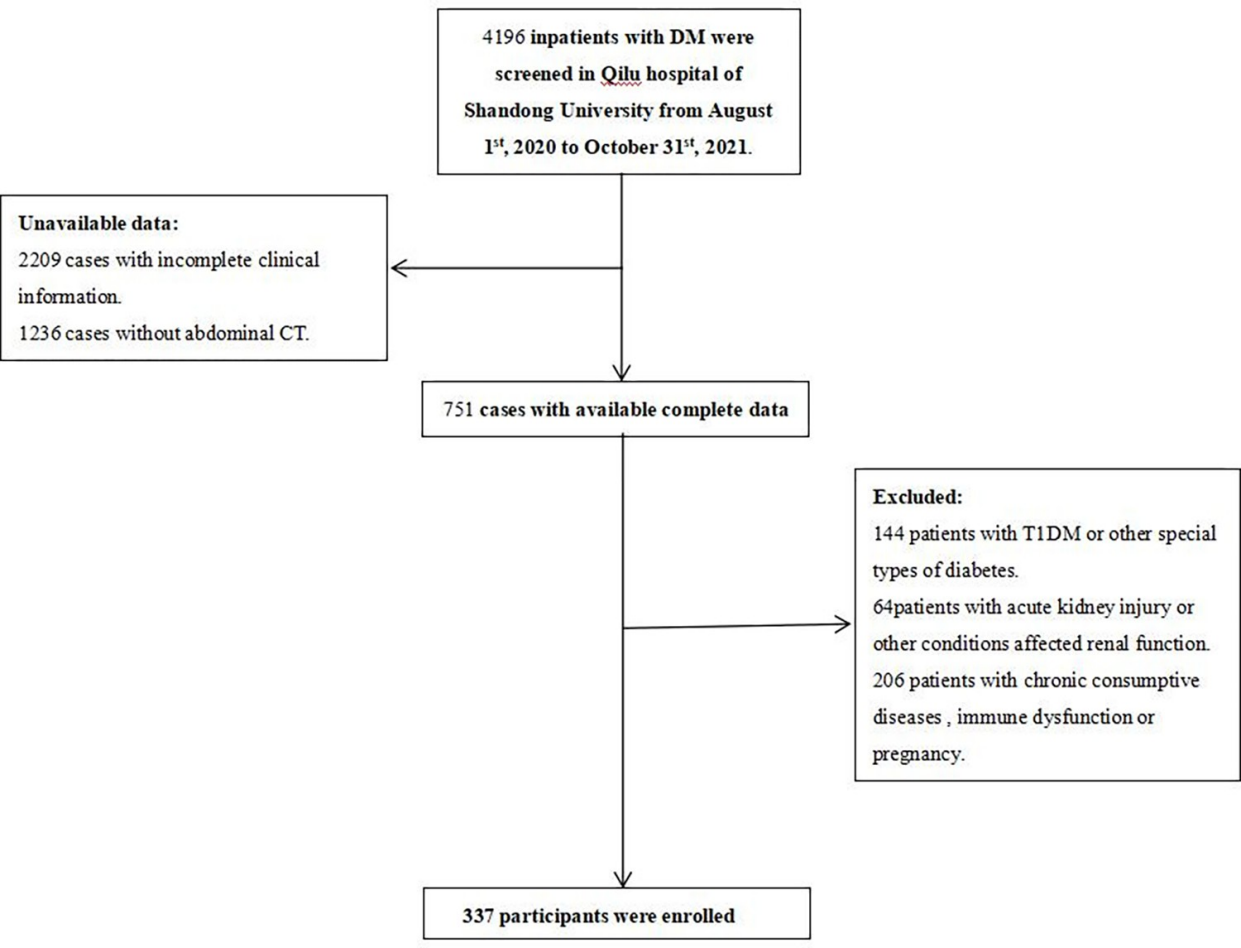
Flowchart of the study population.

This retrospective cross-sectional study was approved by the Ethics Committee on Scientific Research of Shandong University Qilu Hospital (KYLL-202204-031), and informed consent was waived as we only use and report unidentified administrative data. We screened the patients with T2DM admitted at our hospital from August 1^st^, 2020 to October 31^st^, 2021. Patients would be excluded if they have any of the following conditions: (1) renal replacement therapy, such as renal transplantation or renal dialysis; (2) kidney surgery or injury; (3) renal dysfunction caused by renal artery stenosis, renal stones, hydronephrosis, renal atrophy, renal tumor, polycystic kidney, renal developmental abnormalities, infection and inflammatory disease; (4) chronic consumptive diseases; (5) congenital disorders of fat metabolism, including generalized lipodystrophy and Madelung syndrome; (6) Incomplete clinical or imaging data. The screening process for the study population was shown in Fig 1.

### Anthropometric parameter evaluation

The information of subjects was obtained from our case retrieval system. Record the date of T2DM diagnosis, and patients’ height, weight, waist circumference (WC), waist-to-hip ratio (WHR), blood pressure and laboratory data, including fasting plasma glucose (FPG), estimated average glucose (eAG), hemoglobin A1c (HbA1c), total cholesterol (TC), high-density lipoprotein cholesterol (HDL-c), low-density lipoprotein cholesterol (LDL-c), triglycerides (TG), blood urea nitrogen (BUN), Serum creatinine (Scr), free fatty acids (FFA), uric acid (UA), cystatin c (cysc), urinary albumin (Alb), and the urinary albumin creatinine ratio (UACR). The duration of diabetes was days between the date of the original diagnosis and the date of the day’s visit. Height and weight were recorded in light clothing. Body mass index was calculated by dividing weight in kilograms(kg) by the square of height in meters(m). Body surface area was calculated by the DuBois formula (0.20247 * height[m]0.725 * weight[kg]0.425) [14]. Waist circumference was measured at the level of the umbilicus at the end of expiration in the upright position. Waist-to-hip ratio was the ratio of waist circumference to hip circumference. Waist circumference was measured by an inelastic soft ruler in the upright position through the midpoint of the line between the right anterior superior iliac spine and the intersection of the right axillary midline and the 12th rib point in a horizontal direction. The maximum circumference of the most prominent part of the hip was measured as the hip circumference. After resting for at least 5 minutes, an appropriate cuff was used to measure the patient’s blood pressure twice in the sitting position, and take the average value.

The blood biochemical indices were assessed by an automated analyzer (Cobas8000 c701, Roche Diagnostics GmbH, German.) after 8–12 hours of overnight fasting and 15 minutes of rest. Blood samples were stored in a 4–15°C environment and centrifuged for 15 minutes. Urinary albumin and creatinine concentrations were tested by the enzyme-linked immunosorbent assay method (ELISA) using the first sterile urine sample on the morning of the testing day. The urinary albumin creatinine ratio was calculated. eGFR was calculated by the Chronic Kidney Disease Epidemiology Collaboration Equation (CKD-EPI) [21]. Chronic kidney disease (CKD) was defined as eGFR <60 mL/min/1.73m^2^. The intervals of the clinical, laboratory and imaging data are no more than one week.

### Imaging evaluation

All patients underwent CT scanning in the supine position using Force CT (SOMATOM Force, Siemens Healthcare, German). The scanning protocols were as follows: tube voltage: 120 kV; tube current: automatic; beam pitch: 1mm; reconstruction thickness: 1mm; reconstruction interval: 1mm; window width: 260 HU; window level: 50 HU. To ensure reproducibility of the measurements and reliability of the study results, all imaging measurements were performed by two trained radiologists separately (S.N.X., R.C.R.; three years and five years of experience, respectively, who were completely unaware of the subjects’ clinical situation).

**Fig 2:**
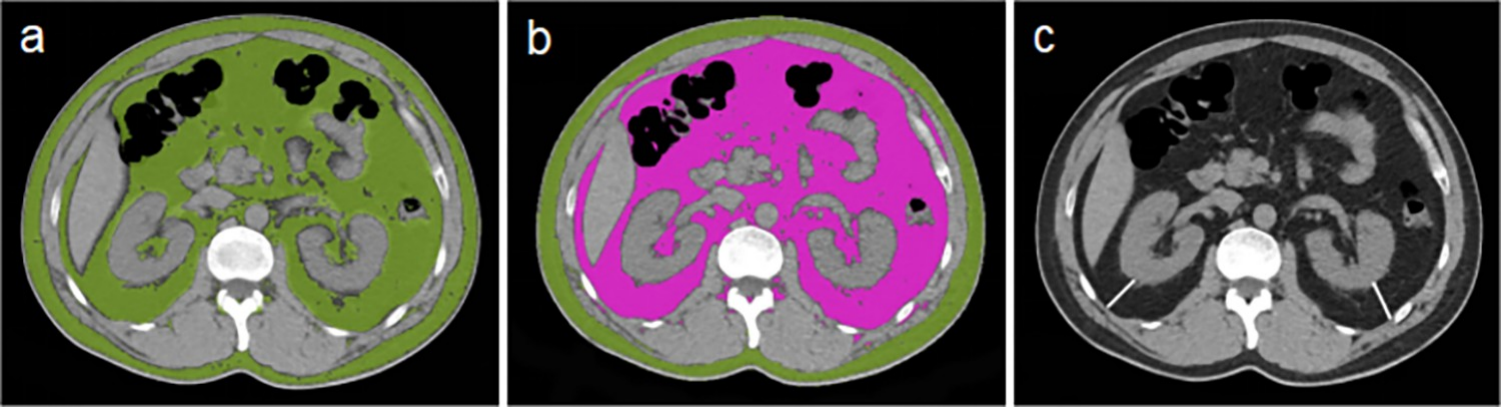
Representative illustrations of fat distribution parameters. a. total abdominal fat (green); b. subcutaneous adipose tissue (green) and visceral adipose tissue (purple); and c. para-perirenal fat thickness (white line).

Total abdominal fat, subcutaneous fat, and visceral fat volumes were segmented automatically on syngo. via post-processing workstation (VB40, Siemens, German). The measurement range was from the top of the diaphragm to the superior margin of the iliac crest, with a fat threshold of -200 ~ -40 HU. The voxels within this CT threshold in the scanning range were defined as total abdominal fat (TAF). The total abdominal fat was divided into internal visceral fat (VAT) and external subcutaneous fat (SAT) by drawing along the ribs and vertebral transverse processes. PRFT was measured along the renal vein extension in the central slice of the renal hilum, as shown in Fig 2C. The distance from the outer border of kidney to the inner border of the abdominal wall muscles was defined as PRFT. PRFT on both sides was measured and the mean PRFT was calculated for subsequent analysis.

### Statistical analysis

Normality tests were performed using the Shapiro-Wilk method. Continuous variables with normal distribution were described as mean ± standard deviation, while those with skewed distribution were described as median (25th percentile, 75th percentile). Categorical variables were expressed as frequencies (proportions). Comparisons between two groups were performed by using Student’s t test, Mann-Whitney U test or chi-square test, as appropriate. Comparisons of multiple groups were performed using One-Way ANOVA and LSD post hoc test or Kruskal-Wallis H. Single-factor correlation was analyzed by using simple linear correlation. Multi-factor linear regression analysis was used to explore the independent influence factors of the outcome variables. The correlation between variables was expressed as pearson or spearman correlation coefficient r and standardized regression coefficient β, respectively. Fisher’s r-to-z transformation was used to compare whether there was a statistical significance between the correlation coefficients. ROC curves were used to assess the diagnostic performance of the models when detecting renal dysfunction in T2DM patients. The AUC of the ROC curves was compared using De Long test. Statistical analysis was performed using the IBM SPSS package (version 23.0 for windows, Chicago, IL), MedCalc statistical software (version 18, Mariakerke, Belgium) and the R statistical package (version 3.5.2, available from http://www.rproject.org).

## Results

### Clinical baseline characteristics of the subjects

This cross-sectional study included 337 subjects with T2DM from a total of 4196 inpatients in the institutional database (195 males, 57.9%; mean age, 60.2 ± 11.6 years). Table 1 showed the clinical characteristics of all subjects and subgroups based on PRFT tertiles. Compared to the low PRFT group (PRFT1), the high PRFT (PRFT3) group was more likely to be male, with greater age, weight, BSA, BMI, WC, WHR, PRFT, TAF, SAT, VAT, SBP, TC, TG, BUN, Scr, UA and cysc, while HDL-c and eGFR were lower in the high PRFT group compared to the low PRFT group (p< 0.001). There was no significant difference in diabetes duration, height, DBP, Ankle-Brachial Index (ABI), eAG, HbA1c, LDL-c, FFA, Alb, or UACR among the three groups (p>0.05). There were more patients with CKD in the high PRFT group than in the low PRFT group (p = 0.001).

**Table 1:** The clinical characteristics of overall populations and subgroups according to the tertiles of the PRFT.

	Overall population n = 337	PRFT1(≤13.3mm) n = 113	PRFT2(13.3–19.9mm) n = 112	PRFT3(>19.9mm) n = 112	P
Male, n (%)	195(57.9)	46(23.6)	69(35.4)	80(41.0)	<0.001
Age(years)	60.2±11.6	57.1±12.0*	60.6±11.9^NS^	62.9±10.2^###^	0.001
Duration of diabetes(years)	11.0(6.0,20.0)	10.0(6.0,16.5)	10.0(6.0,20.0)	13.0(6.7,20.0)	>0.05
FPG(mmol/L)	7.8(6.7,8.1)	7.8(6.4,7.8)**	7.8(6.8,9.0)^NS^	7.8(6.6,7.8)^NS^	<0.01
Height(cm)	166.7±8.2	165.2±8.1	167.5±8.1	167.5±8.2	>0.05
Weight(kg)	71.4±11.8	64.8±9.9***	72.3±10.6^^^^	77.2±11.4^###^	<0.001
BSA	1.80±0.17	1.71±0.15***	1.81±0.16^^^	1.86±0.17^###^	<0.001
BMI(kg/m^2^)	25.7±3.5	23.7±3.3***	25.9±2.8^^^^^	27.6±3.3^###^	<0.001
WC(cm)	93.7±9.6	87.9±8.7***	94.8±7.7^^^^	98.6±9.0^###^	<0.001
WHR	0.94±0.06	0.91±0.05***	0.95±0.05^NS^	0.96±0.06^###^	<0.001
PRFT(mm)					
LEFT	16.1(11.1,21.8)	9.4(6.5,11.1)***	16.2(14.4,18.1)^^^^^	24.9(21.8,28.7)^###^	<0.001
RIGHT	16.3(11.5,22.4)	9.3(7.1,11.6)***	16.3(14.8,18.2)^^^^^	25.0(22.4,28.7)^###^	<0.001
MEAN	16.1(11.2,22.0)	9.3(7.0,11.2)***	16.1(14.6,18.1)^^^^^	25.1(21.9,28.9)^###^	<0.001
TAF(cm3)	5775.5(4085.0,7439.5)	4064.2(2742.7,5669.4)***	5603.6(4580.9,7336.0)^^^^^	7222.0(6088.4,8488.2)^###^	<0.001
SAT(cm3)	2576.9(1880.0,3627.4)	2328.1(1451.3,3200.1)^NS^	2469.3(1865.6,3675.1)^NS^	2918.2(2232.5,3774.6)^###^	<0.001
VAT(cm3)	2850.7(1899.5,4043.5)	1693.8(1079.1,2509.5)***	2986.9(2296.3,3707.2)^^^^^	4278.8(3266.8,4850.2)^###^	<0.001
SBP(mmHg)	137.0(124.0,152.5)	132.0(120.0,144.0)*	139.5(127.0,155.0)^NS^	138.1(125.0,158.8)^#^	<0.01
DBP(mmHg)	78.0(70.0,86.0)	77.0(67.5,86.0)	78.0(71.3,85.0)	78.3(71.0,88.0)	>0.05
ABI					
LEFT	1.15(1.07,1.25)	1.14(1.06,1.25)	1.15(1.07,1.25)	1.17(1.08,1.26)	>0.05
RIGHT	1.15(1.08,1.24)	1.15(1.06,1.24)	1.15(1.08,1.23)	1.15(1.10,1.25)	>0.05
eAG(mmol/L)	11.5(8.6,13.7)	11.5(8.6,15.0)	11.3(8.6,13.0)	10.5(8.5,13.0)	>0.05
HbA1c(%)	8.3(6.8,9.5)	8.5(6.8,10.2)	8.3(6.8,9.3)	7.8(6.7,9.1)	>0.05
TC(mmol/L)	4.3(3.6,5.1)	4.4(3.9,5.2)^NS^	4.4(3.6,5.0)^NS^	4.1(3.2,4.8)^#^	0.042
HDL-c(mmol/L)	1.15±0.30	1.25±0.38**	1.14±0.27^^^	1.05±0.21^###^	<0.001
LDL-c(mmol/L)	2.44(1.82,3.04)	2.54(2.11,3.12)	2.45(1.75,3.06)	2.31(1.72,2.96)	>0.05
TG(mmol/L)	1.34(0.95,1.90)	1.14(0.83,1.69)*	1.48(1.03,1.93)^NS^	1.50(1.02,2.04)^##^	<0.01
BUN	5.5(4.53,6.74)	5.40(3.97,6.70)^NS^	5.45(4.50,6.40)^^^	5.92(4.82,7.25)^#^	<0.01
Scr(umol/L)	68.0(56.0,77.5)	58.0(49.0,70.0)***	70.0(60.0,77.75)^NS^	73.84(62.25,85.75)^###^	<0.001
FFA(umol/dL)	50.0(33.5,65.2)	50.0(35.0,64.8)	48.85(29.5,62)	50.0(35.25,70.53)	>0.05
UA(umol/L)	294.2(237.0,334.5)	282.0(223.5,305.5)^NS^	294.2(233.3,338.0)^NS^	294.2(257.5,350.75)^#^	<0.05
cysc	0.98(0.83,1.11)	0.88(0.79,1.06)*	1.00(0.83,1.11)^NS^	1.05(0.91,1.24)^###^	<0.001
Alb	13.09(6.80,72.14)	12.62(6.63,52.37)	13.89(6.73,79.9)	12.52(7.33,86.11)	>0.05
UACR(mg/g)	0.02(0.01,0.14)	0.02(0.01,0.10)	0.02(0.01,0.17)	0.02(0.01,0.17)	>0.05
eGFR(ml/min/1.73m^2^)-EPI	93.5(83.9,104.49)	100.95(90.91,108.12)***	90.91(82.62,100.50)^NS^	90.92(72.35,100.43)^###^	<0.001
eGFR(ml/min/1.73m^2^)-EPI					
CKD (eGFR<60)	29(8.6)	4(3.5)	6(5.4)	19(17.0)	0.001
non-CKD(eGFR≥60)	308(91.4)	109(96.5)	106(94.6)	93(83))	0.001

Note.- Continuous variables are expressed as mean ± standard deviation or median (interquartile) unless otherwise specified. Categorical variables are absolute numbers (relative numbers). FPG = fast plasma glucose. BSA = body surface area. BMI = body mass index. WC = waist circumference. WHR = waist-to-hip ratio. PRFT = para-perirenal fat thickness. TAF = total abdominal fat. SAT = subcutaneous adipose tissue. VAT = visceral adipose tissue. SBP = systolic blood pressure. DBP = diastolic blood pressure. ABI = ankle-brachial index. eAG = estimated average glucose. HbA1c = glycated hemoglobin. TC = total cholesterol. HDL-c = high-density lipoprotein-cholesterol. LDL-c = low-density lipoprotein-cholesterol. TG = triglyceride. BUN = blood urea nitrogen. Scr = serum creatinine. FFA = free fatty acid. UA = uric acid. Alb = albumin. UACR = urinary albumin creatinine ratio. eGFR = estimated glomerular filtration rate. CKD = chronic kidney disease.

PRFT1 vs. PRFT2: *P < 0.05, **P < 0.01,***P<0.001

PRFT1 vs. PRFT3: ^#^P < 0.05, ^##^P < 0.01, ^###^P<0.001

PRFT2 vs. PRFT3: ^^^P < 0.05, ^^^^P < 0.01,^^^^^P<0.001

Table 2 demonstrated the sex differences in each obesity-related indicator. Females were older than males (p = 0.001, 95% CI = 1.68–6.65). Men had higher height, weight, BSA, WC, WHR, PRFT, VAT and eGFR than women (p<0.05). However, men had smaller SAT than women (p<0.001). There was no statistical difference in duration, BMI, TAF and the presence of CKD between men and women (p >0.05). Patients with CKD had higher BMI, WC, WHR, TAF, VAT, and PRFT compared to those without CKD. There was no difference in BSA and SAT between CKD and non-CKD group (p>0.05) (S1 Table).

**Table 2:** Comparison of obesity-related indicators and eGFR grouped by gender.

	Man n = 195	Woman n = 142	P
Age(years)	58.5±12.2	62.6±10.4	0.001
Duration of diabetes(years)	10.0(5.00,20.0)	12.5(7.00,20.0)	>0.05
Height(cm)	171.8±5.9	159.8±5.2	<0.001
Weight(kg)	75.9±10.6	65.2±10.5	<0.001
BSA	1.88±0.14	1.68±0.13	<0.001
BMI(kg/m^2^)	25.8±3.0	25.6±4.1	>0.05
WC(cm)	95.5±8.4	91.3±10.6	<0.001
WHR	0.95±0.60	0.92±0.51	<0.01
PRFT(mm)			
LEFT	18.20(12.80,23.80)	13.10(8.55,18.49)	<0.001
RIGHT	18.50(13.65,24.15)	13.85(8.70,18.36)	<0.001
MEAN	18.38(13.58,23.95)	14.04(8.50,18.39)	<0.001
TAF(cm3)	5894.2(4095.8,7294.6)	5669.4(4081.3,7583.9)	>0.05
SAT(cm3)	2254.2(1625.3,2977.6)	3256.8(2305.2,4243.7)	<0.001
VAT(cm3)	3398.0(2356.7,4473.1)	2358.4(1602.9,3281.1)	<0.001
eGFR(ml/min/1.73m^2^)-EPI	96.4(87.2,105.5)	90.9(77.3,101.1)	0.002
eGFR(ml/min/1.73m^2^)-EPI			
CKD (eGFR<60)	16(8.2)	13(9.2)	>0.05
non-CKD(eGFR≥60)	179(91.8)	129(90.8)	

Note.- Continuous variables are expressed as mean ± standard deviation or median (interquartile) unless otherwise specified. Categorical variables are absolute numbers (relative numbers). BSA = body surface area. BMI = body mass index. WC = waist circumference. WHR = waist-to-hip ratio. PRFT = para-perirenal fat thickness. TAF = total abdominal fat. SAT = subcutaneous adipose tissue. VAT = visceral adipose tissue. eGFR = estimated glomerular filtration rate. CKD = chronic kidney disease.

### Correlation of anthropometric parameters with obesity-related indicators

**Fig 3:**
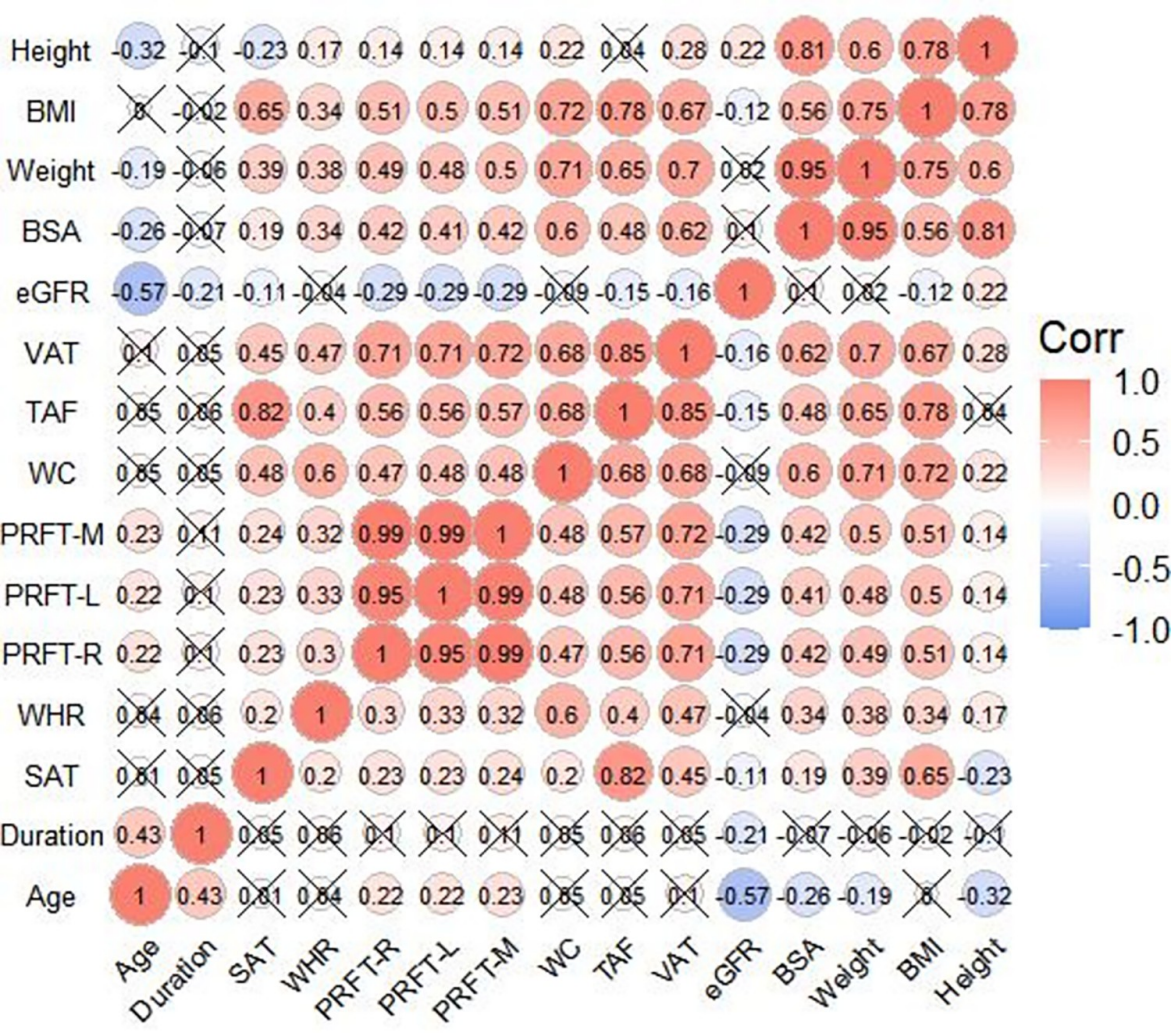
Correlation heatmap of each parameter of anthropometric and obesity-related indicators. The numbers and color in the circles indicated the correlation coefficient (r), and the wrong symbols indicated no statistical significance, otherwise it was statistically significant.

The correlation coefficient of the left and right PRFT was 0.951. The inter-classic correlation coefficients (ICC) of the average PRFT, TAF, SAT, and VAT were 0.827, 0.999, 0.998 and 0.997, respectively. Fig 3 showed the correlation of anthropometric parameters and each obesity-related indicator in the total population. All obesity-related indicators were significantly correlated with each other (p<0.001). Age showed a significant negative correlation with height, weight and BSA (p<0.001), whereas it was positively correlated with the duration of disease and PRFT. In addition, the duration of the disease was found to be independent of any obesity-related indicators (p>0.05). Height was not correlated to TAF(r = 0.041, p>0.05), but significantly negatively correlated with SAT (r = -0.232, p<0.001) and significantly positively correlated with weight, BSA, BMI, WC, WHR, PRFT, VAT (all p<0.05). Weight was significantly positively correlated with all obesity-related indicators (all p<0.001). eGFR was not correlated with weight, WC or WHR (r = 0.017, 0.099, -0.087, p = 0.749, 0.070, 0.500, respectively). Moreover, eGFR was not correlated with BSA (r = -0.037, p = 0.110), which was in agreement with the fact that CKD-EPI formula has adjusted eGFR by a BSA of 1.73m^2^. However, eGFR was correlated with age, duration of disease, height, BMI, PRFT, TAF, SAT, and VAT. Among them, age and PRFT had the strongest correlation with eGFR (r = -0.573 vs. -0.294, both p<0.001).

**Fig 4:**
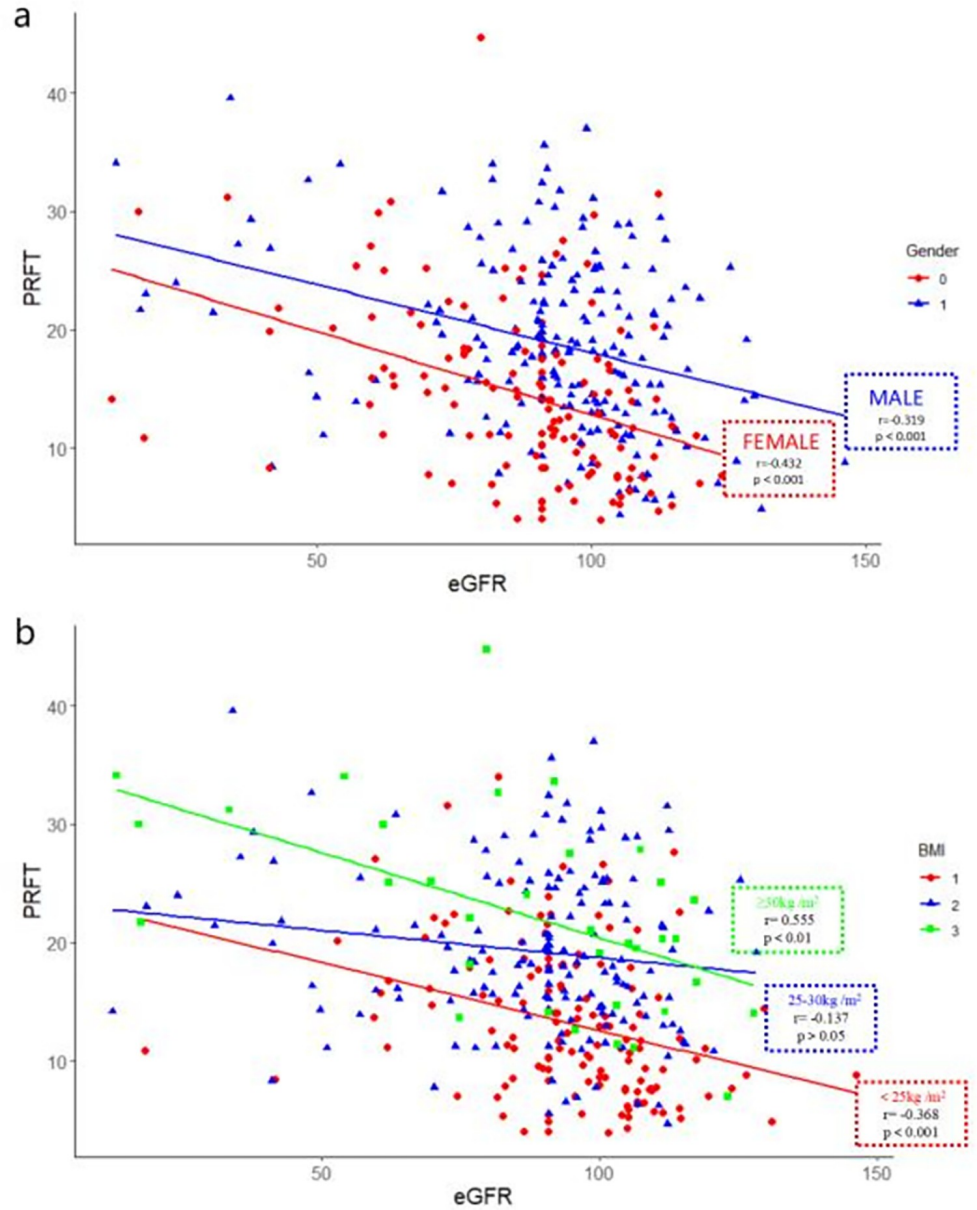
The correlations between eGFR and PRFT in the study population. The correlations between eGFR and PRFT in the subgroups divided by sex (a) and BMI (b), respectively.

Table 3 showed the relationship between eGFR and obesity-related indicators in subgroups separated by sex. eGFR was significantly and negatively correlated to age and PRFT both in male and female subgroup (p<0.001). Moreover, eGFR was also negatively correlated to duration of disease in men (r = -0.231, p = 0.001) and in women(r = 0.175, p = 0.037). eGFR was positively correlated with height in men (r = 0.198, p = 0.001) and negatively correlated with weight in women (r = -0.169, p<0.05). eGFR was not correlated to BSA or WHR in two subgroups. Moreover, eGFR was negatively correlated with BMI, WC, TAF, and VAT in women (p<0.05). S2 Table showed the relationship between eGFR and obesity-related indicators in subgroups according to BMI. eGFR showed a significant negative correlation with age (p<0.001) and a positive correlation with height in three groups (p<0.05). eGFR was positively correlated with BSA in the overweight and obese groups while it had a negative correlation with VAT in the normal group (r = -0.216, p = 0.010). eGFR was significantly negatively correlated with duration in the overweight group (r = -0.274, p<0.001). It was worth noting that eGFR was significantly negatively correlated with PRFT in the normal and obese groups, while it was not correlated to PRFT in the overweight group, which may be due to the small subgroup after slicing and dicing. All three groups showed no significant relationship with the rest of the obesity-related indicators. None of the correlation coefficients reached statistical significance in different subgroups based on gender and BMI (p>0.05) (Fig 4). S3 Table demonstrated the relationship between eGFR and obesity-related indicators in the subgroups of CKD and non-CKD. Interestingly, eGFR was positively correlated with BSA in T2DM patients with non-CKD (r = 0.201, p<0.001), but negatively correlated with BSA in patients with CKD (r = -0.418, p<0.05). eGFR was significantly negatively correlated with PRFT in subjects with non-CKD (r = -0.250, p<0.001), but it was not related to PRFT in patients with CKD (r = -0.218, p>0.05), which was likely due to a small sample in CKD group. In addition, eGFR was negatively correlated with BMI, WC, WHR, and VAT in CKD group (all p<0.01).

**Table 3:** Main correlations of anthropometric and eGFR in the subgroup divided by sex.

Parameter	Men(n = 195)		Women(n = 142)	
	r	P	r	P
Age(years)	-0.578	0.000	-0.544	0.000
Duration of diabetes(years)	-0.231	0.001	-0.175	0.037
Height(cm)	0.198	0.006	0.103	0.223
Weight(kg)	0.011	0.884	-0.169	0.044
BSA	0.095	0.189	-0.157	0.067
BMI(kg/m^2^)	-0.078	0.277	-0.181	0.031
WC(cm)	-0.041	0.570	-0.251	0.003
WHR	-0.031	0.668	-0.156	0.065
PRFT(mm)				
LEFT	-0.315	0.000	-0.417	0.000
RIGHT	-0.308	0.000	-0.439	0.000
MEAN	-0.319	0.000	-0.432	0.000
TAF(cm3)	-0.070	0.332	-0.247	0.003
SAT(cm3)	-0.008	0.912	-0.145	0.085
VAT(cm3)	-0.133	0.064	-0.354	0.000

Note.- BSA = body surface area. BMI = body mass index. WC = waist circumference. WHR = waist-to-hip ratio. PRFT = para-perirenal fat thickness. TAF = total abdominal fat. SAT = subcutaneous adipose tissue. VAT = visceral adipose tissue.

### Multi-factor analysis after correcting for confounding variables

**Fig 5:**
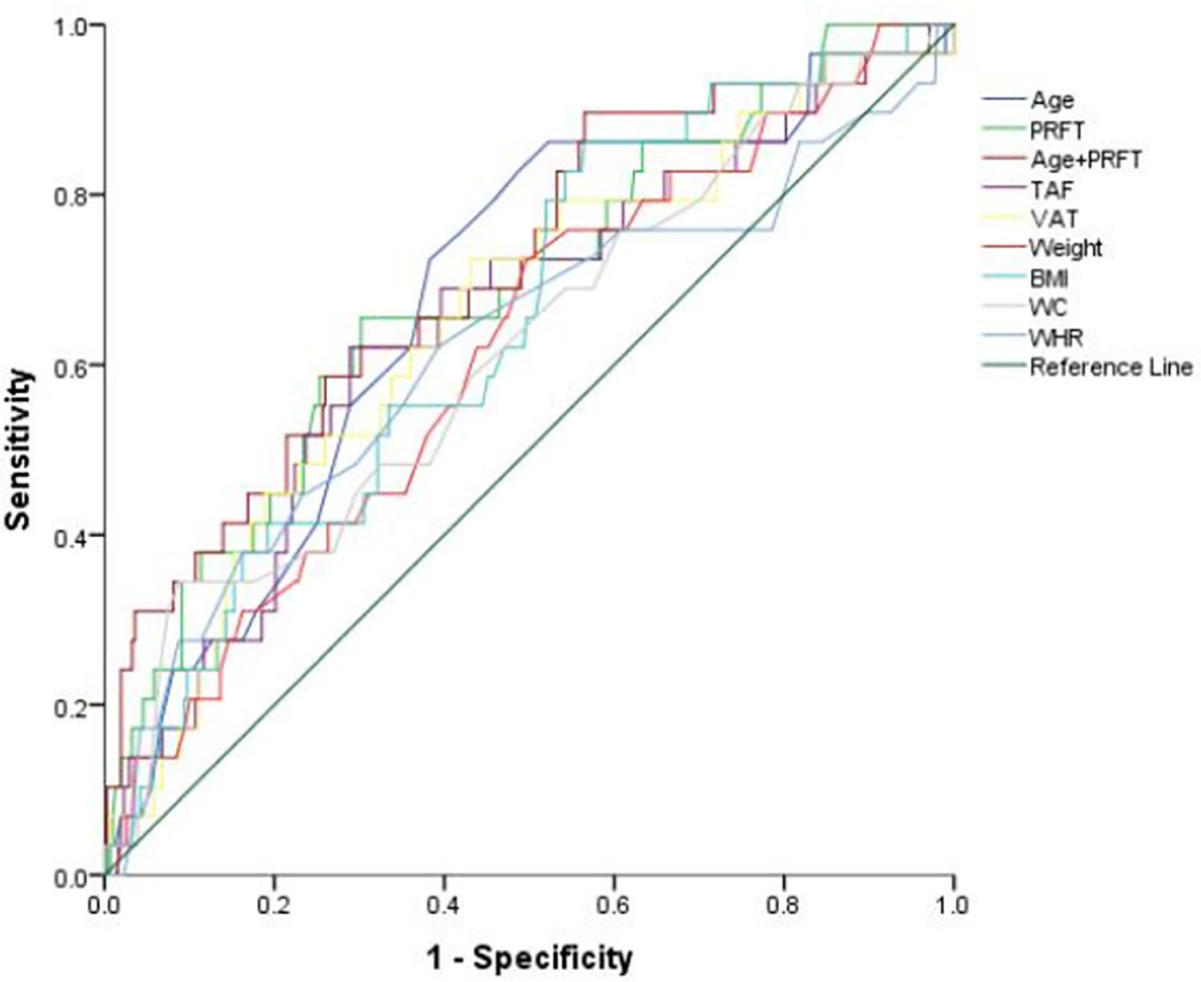
Receiver operating characteristic (ROC) curves for detection of chronic kidney disease (CKD) in T2DM. ROC curves of para‐perirenal fat thickness (PRFT) and other obesity-related indicators for detection of estimated glomerular filtration rate (eGFR) <60 mL/min per 1.73 m^2^.

The eGFR was only influenced by age and PRFT independently in male and female, as shown in Table 4. Fig 5 showed the ROC curve to assess the performance of obesity-related indicators and joint model in diagnosing CKD. Table 5 showed the diagnostic performance parameters of each model. Among those obesity-related indicators, PRFT had the best diagnostic performance (AUC = 0.686, p = 0.001, 95% CI = 0.582–0.791). The best cutoff of PRFT that could better distinguish normal renal function from low eGFR in T2DM patients was 19.85 mm, with sensitivity and specificity of 65.52% and 69.81%, respectively. The positive predictive value was 17.0% and the negative predictive value was 95.6%. Therefore, the probability of normal kidney in T2DM patients with PRFT ≤ 19.85 mm was 95.6%. The largest AUC was obtained by uniting age and PRFT (AUC = 0.708, p<0.001, 95%CI = 0.604–0.812). However, none of them reached statistical significance compared to PRFT in predicting CKD in patients with T2DM. In addition, height, BSA and SAT have no predictive value for CKD in T2DM.

**Table 4:** Independent multivariate correlates of eGFR.

Parameter	Unstandardized coefficient(B)	Standardized coefficient(β)	P	95%CI
Model: male(adjusted R^2^ = 0.232)				
PRFT(mm)	-0.624	-0.215	0.001	-1.002 - -0.246
Age(year)	-0.626	-0.358	<0.001	-0.899 - -0.352
Model:female(adjusted R^2^ = 0.263)				
PRFT(mm)	-0.602	-0.217	0.039	-1.173 - -0.032
Age(year)	-0.665	-0.342	<0.001	-1.022- -0.308

Note.- Multivariate linear regression model after correcting for age, duration of illness, height, weight, BMI, WC, TAF, VAT. PRFT = para-perirenal fat thickness

**Table 5:** Diagnostic performance parameters of the ROC curve.

	AUC	SE	P	95%CI
Age	0.676	0.051	0.002	0.577–0.775
PRFT	0.686	0.053	0.001	0.582–0.791
Age+PRFT	0.708	0.053	<0.001	0.604–0.812
TAF	0.650	0.055	0.008	0.543–0.758
VAT	0.654	0.054	0.006	0.548–0.760
Weight	0.616	0.053	0.039	0.511–0.720
BMI	0.648	0.050	0.009	0.549–0.746
WC	0.619	0.057	0.034	0.508–0.730
WHR	0.618	0.061	0.036	0.497–0.738

Note.- PRFT = para-perirenal fat thickness. TAF = total abdominal fat. VAT = visceral adipose tissue. BMI = body mass index. WC = waist circumference. WHR = waist-to-hip ratio.

## Discussion

Numerous studies have shown that obesity causes fat deposition around organs and organ dysfunction [22–25]. In 1974, Weisinger et al. linked obesity to proteinuria first [26]. Subsequently, several studies showed that visceral fat was truly correlated to renal dysfunction and cardiometabolism [9, 27].

In our study, we demonstrated that eGFR was correlated strongly with age and PRFT in T2DM as well as in the subgroups separated by sex. PRFT, as one of the independent risk factors of CKD, had a better diagnostic performance than other obesity-related indicators. This phenomenon may be explained by the inherent limitations of traditional indicators [11, 12]. For BMI and WC, it is difficult to distinguish fat from other tissue components or differentiate subcutaneous fat from visceral fat [11, 12]. In addition, PRFT is superior to VAT in the diagnosis of CKD in T2DM for several possible reasons. Firstly, perirenal fat locating around the kidney is the only adipose tissue encapsulated by connective tissue [17]. As soon as the perirenal fat expands, it will compress the kidney directly and cause decreased renal blood supply and increased interstitial hydrostatic pressure which impairs renal function [20, 28, 29]. Lamacchia et al. found that PRFT was an independent predictor for CKD, increased renal resistance index, and hyperuricemia in T2DM [20]. Secondly, perirenal fat, as a special visceral fat, has an intact vascular, neurological, and lymphatic system [30–32], which may cause systemic renal damage different from other visceral adipose tissues. Thirdly, perirenal fat is a special endocrine organ that secretes bioactive substances such as cytokines and adipokines, which affect renal function through paracrine or endocrine mechanisms [33–35]. The study by Li et al. indicated that perirenal fat can produce leptin, which led to glomerular endothelial cell proliferation through activation of the p38 MAPK pathway [33]. Another study showed that TNF-α released from perirenal fat caused endothelial cell dysfunction in renal arteries [34]. In addition, perirenal fat can secrete FFA into the kidney. Increased intracellular fatty acid metabolites can lead to renal impairment [35]. At last, there are different origins, developmental genes and expression patterns in visceral fat and subcutaneous fat [36, 37], which may explain the fact that perirenal fat is more likely to impact renal function compared to other kinds of fats.

A cross-sectional study of risk factors for CKD in T2DM by Chen X et al. concluded that lower eGFR was correlated to higher PRFT [13]. The multiple linear regression analysis adjusted for confounding factors showed that PRFT was independently and negatively correlated to eGFR [13], which was consistent with our findings. However, the study did not conclude that eGFR was correlated to TAF, SAT, and VAT. A study by Fang Y et al. demonstrated a significantly negative correlation between eGFR and PRFT in the total population and men, whereas there was no significant relationship between the two variables in women [19]. These inconsistencies may be due to the different characteristics of the subjects recruited.

As reported that CT was the gold standard to measure the volume of adipose tissue [38, 39], CT was used to evaluate PRFT, TAF, SAT, and VAT in the large population study. To our knowledge, the evaluations of fat content were based on dual energy X-ray absorptiometer (DXA) mainly in previous studies [13], and some researches assessed the fat area rather than volume via bioelectrical impedance analysis (BIA) [19]. So, the application of CT in evaluating the fat volume was novel. Moreover, we provided a comprehensive and comparative evidence about the predictive value of all obesity-related indicators for CKD in T2DM and developed a joint diagnostic model by linking age and PRFT to assess CKD. Our research showed that para-perirenal fat thickness (the distance from the outer border of kidney to the inner border of the abdominal wall muscles on the extension of the renal vein) measured by CT at the level of the renal hilum provided a simple and reliable estimate for perirenal fat volume.

There were some limitations in our study. Firstly, due to the retrospective nature, there may be a potential for selection bias in this study, though we fully considered the situations that might influence the screening of study population and selected subjects strictly according to the inclusion-exclusion criteria. Our study was a cross-sectional research. It could only propose a hypothesis rather than establish a causal relationship between PRFT and eGFR. It had been reported that CKD may lead to the redistribution of adipose tissue, causing ectopic fat deposition in the liver, kidneys, and other organs [35]. So it was also possible that increased PRFT is caused by CKD. Thus, more prospective or experimental studies with a larger study population would be warranted. Secondly, we used estimated GFR calculated by the EPI formula rather than measured GFR, which was the most accurate evaluation of renal function. Thirdly, our study only investigated the relationship between obesity-related indicators and renal function in T2DM. We did not pay attention to dietary, lifestyle or biochemistry indicators, which are potential factors that affect eGFR [14, 40]. At last, this study was designed as a single center. Therefore, the generalization of our results required further external validation.

In conclusion, the combined diagnostic model linking age to PRFT has an excellent performance for the assessment of renal impairment in T2DM. In addition, those variables including VAT, TAF and BMI have a nice diagnostic value only second to age and PRFT.

## Supporting information

S1 Checklist STROBE statement—Checklist of items that should be included in reports of *cross-sectional studies*. (DOCX)

S1 Table Comparison of obesity-related parameters between the chronic kidney disease and non-chronic kidney disease. (DOCX)

S2 Table Main correlations of anthropometric and eGFR in the subgroup divided by BMI. (DOCX)

S3 Table Main correlations of anthropometric and eGFR in the subgroup divided by whether suffered from CKD. (DOCX)

S1 Data set (XLSX)

## Abbreviations

BSA: Body surface area
BMI: Body mass index
WC: Waist circumference
WHR: Waist-to-hip ratio
PRFT: Para-perirenal fat thickness
TAF: Total abdominal fat
SAT: Subcutaneous adipose tissue
VAT: Visceral adipose tissue
eGFR: estimated glomerular filtration rate
CKD: Chronic kidney disease
