# Supplementary material for: Para-perirenal fat thickness is associated with reduced glomerular filtration rate regardless of other obesity-related indicators in patients with type 2 diabetes mellitus
Source: PLoS One. 2023 Oct 26;18(10):e0293464. doi: 10.1371/journal.pone.0293464 (PMC10602252; doi:10.1371/journal.pone.0293464)
Supplement: S1 Table — (DOCX) [file pone.0293464.s002.docx]

**S1 Table.**

|  | Non-CKD(n=308) | CKD(n=29) | P |
| --- | --- | --- | --- |
| BSA | 1.79±0.17 | 1.84±0.17 | >0.05 |
| BMI(kg/m2) | 25.55±3.49 | 27.32±3.46 | <0.01 |
| WC(cm) | 93.34±9.37 | 97.81±10.78 | <0.05 |
| WHR | 0.94±0.06 | 0.96±0.07 | <0.05 |
| TAF (cm3) | 5638.37(4045.41,7254.95) | 7336.42(5085.80,8585.22) | <0.01 |
| SAT (cm3) | 2555.44(1838.35,3556.63) | 2834.95(2213.18,4409.33) | >0.05 |
| VAT(cm3) | 2792.62(1873.45,3890.20) | 3761.82(2726.61,4688.95) | <0.01 |
| PRFT(mm)  LEFT  RIGHT  MEAN | 15.88(10.91,21.35)  16.05(11.26,21.65)  15.81(11.08,21.29) | 20.85(13.7,28.28)  22.75(14.63,30.90)  21.68(14.26,28.26) | <0.01  <0.01  <0.01 |

Note.- BSA = body surface area. BMI = body mass index. WC = waist circumference. WHR = waist-to-hip ratio. PRFT = para-perirenal fat thickness. TAF = total abdominal fat. SAT = subcutaneous adipose tissue. VAT = visceral adipose tissue.
