# Supplementary material for: Para-perirenal fat thickness is associated with reduced glomerular filtration rate regardless of other obesity-related indicators in patients with type 2 diabetes mellitus
Source: PLoS One. 2023 Oct 26;18(10):e0293464. doi: 10.1371/journal.pone.0293464 (PMC10602252; doi:10.1371/journal.pone.0293464)
Supplement: S2 Table — (DOCX) [file pone.0293464.s003.docx]

**S2 Table.**

| Parameter | Normal(BMI＜25)142 | | Overweight（BMI25-29.9）161 | | Obesity  （BMI≥30）34 | |
| --- | --- | --- | --- | --- | --- | --- |
|  | r | P | r | P | r | p |
| Age(years) | -0.510 | 0.000 | -0.555 | 0.000 | -0.679 | 0.000 |
| Duration of diabetes(years) | -0.122 | 0.150 | -0.274 | 0.000 | -0.312 | 0.073 |
| Height(cm) | 0.243 | 0.004 | 0.157 | 0.047 | 0.543 | 0.001 |
| Weight(kg) | 0.140 | 0.098 | 0.136 | 0.085 | 0.301 | 0.084 |
| BSA | 0.151 | 0.075 | 0.160 | 0.047 | 0.387 | 0.024 |
| BMI(kg/m²) | -0.153 | 0.070 | -0.019 | 0.811 | -0.279 | 0.110 |
| WC(cm) | -0.059 | 0.486 | -0.022 | 0.781 | -0.055 | 0.756 |
| WHR | 0.066 | 0.436 | 0.041 | 0.609 | -0.155 | 0.383 |
| PRFT(mm)  LEFT  RIGHT  MEAN | -0.372  -0.361  -0.368 | 0.000  0.000  0.000 | -0.127  -0.133  -0.137 | 0.109  0.092  0.084 | -0.533  -0.552  -0.555 | 0.001  0.001  0.001 |
| TAF(cm3) | -0.180 | 0.032 | -0.069 | 0.387 | -0.184 | 0.297 |
| SAT(cm3) | -0.075 | 0.376 | -0.061 | 0.441 | -0.160 | 0.367 |
| VAT(cm3) | -0.216 | 0.010 | -0.064 | 0.422 | -0.066 | 0.711 |

Note.- BSA = body surface area. BMI = body mass index. WC = waist circumference. WHR = waist-to-hip ratio. PRFT = para-perirenal fat thickness. TAF = total abdominal fat. SAT = subcutaneous adipose tissue. VAT = visceral adipose tissue.
