# Supplementary material for: Para-perirenal fat thickness is associated with reduced glomerular filtration rate regardless of other obesity-related indicators in patients with type 2 diabetes mellitus
Source: PLoS One. 2023 Oct 26;18(10):e0293464. doi: 10.1371/journal.pone.0293464 (PMC10602252; doi:10.1371/journal.pone.0293464)
Supplement: S3 Table — (DOCX) [file pone.0293464.s004.docx]

**S3 Table.**

|  | Non-CKD(n=308) | | | CKD(n=29) | |
| --- | --- | --- | --- | --- | --- |
|  | r | p | r | | p |
| BSA | 0.201 | 0.000 | -0.418 | | 0.024 |
| BMI(kg/m2) | -0.064 | 0.263 | -0.488 | | 0.007 |
| WC(cm) | -0.048 | 0.397 | -0.541 | | 0.002 |
| WHR | 0.056 | 0.323 | -0.534 | | 0.003 |
| TAF (cm3) | -0.092 | 0.106 | -0.298 | | 0.116 |
| SAT (cm3) | -0.078 | 0.173 | -0.089 | | 0.647 |
| VAT(cm3) | -0.099 | 0.082 | -0.494 | | 0.007 |
| PRFT(mm)  LEFT  RIGHT  MEAN | -0.252  -0.241  -0.250 | 0.000  0.000  0.000 | -0.177  -0.219  -0.218 | | 0.357  0.253  0.255 |

Note.- BSA = body surface area. BMI = body mass index. WC = waist circumference. WHR = waist-to-hip ratio. PRFT = para-perirenal fat thickness. TAF = total abdominal fat. SAT = subcutaneous adipose tissue. VAT = visceral adipose tissue.
